# Fun and Farming

**Published:** 1871-10

**Authors:** 


### Fun and Farming.

The editor of the Trenton (N. J.) *Sentinel* is telling what *he* "knows about farming," *a la* Greeley. The following is a part of his first essay on the subject:—

Castor oil beans succeed best in the bowels of the earth. They will soon work their way out.

The best preparation for hops, is a toad or two in each hill. They will make the vines fairly jump.

The usual time for putting in rye is early in the morning. Some husbandmen, especially those of the city, continue to run it in at intervals of half an hour, until bed-time. The practice is only allowable in case of a dry season.

In reaping wheat, never take it by the beard. It is found to go against the grain.

Corn in the ear is apt to affect the hearing. If eaten green, it will make the voice husky When dealt out as army rations, the kerne. should be served first, and then the men, pri-vate-ly.

Never plant your potatoes early. It is the early potato that gets the worm. It is up-hill work with them after that.

In making cider out of apples, I found it a pretty tight squeeze, notwithstanding my long connection with the press. Never drink cider made from crab apples. It is pretty certain to go "back on you." If you would lay in a supply of old wine, be sure to make it of elderberries.

---

ALCOHOL AND EYESIGHT.—M. Galezowski recently, at a sitting of the Paris Academy of Medicine, pointed out how many cases he had seen among the poorer classes of loss of vision from the chronic use of alcohol. The form of loss of sight is that of amblyopia.

